# Cloning, Assembly, and Modification of the Primary Human Cytomegalovirus Isolate Toledo by Yeast-Based Transformation-Associated Recombination

**DOI:** 10.1128/mSphereDirect.00331-17

**Published:** 2017-10-04

**Authors:** Sanjay Vashee, Timothy B. Stockwell, Nina Alperovich, Evgeniya A. Denisova, Daniel G. Gibson, Kyle C. Cady, Kristofer Miller, Krishna Kannan, Daniel Malouli, Lindsey B. Crawford, Alexander A. Voorhies, Eric Bruening, Patrizia Caposio, Klaus Früh

**Affiliations:** a J. Craig Venter Institute, Rockville, Maryland, USA; b Synthetic Genomics Inc., La Jolla, California, USA; c Tomegavax Inc., Beaverton, Oregon, USA; d Vaccine and Gene Therapy Institute, Oregon Health & Science University, Beaverton, Oregon, USA; University of Michigan—Ann Arbor; FORGE Life Science; Cardiff University

**Keywords:** Saccharomyces cerevisiae, cloning, cytomegalovirus, genetic recombination

## Abstract

Genetic engineering of cytomegalovirus (CMV) currently relies on generating a bacterial artificial chromosome (BAC) by introducing a bacterial origin of replication into the viral genome using *in vivo* recombination in virally infected tissue culture cells. However, this process is inefficient, results in adaptive mutations, and involves deletion of viral genes to avoid oversized genomes when inserting the BAC cassette. Moreover, BAC technology does not permit the simultaneous manipulation of multiple genome loci and cannot be used to construct synthetic genomes. To overcome these limitations, we adapted synthetic biology tools to clone CMV genomes in Saccharomyces cerevisiae. Using an early passage of the human CMV isolate Toledo, we first applied transformation-associated recombination (TAR) to clone 16 overlapping fragments covering the entire Toledo genome in Saccharomyces cerevisiae. Then, we assembled these fragments by TAR in a stepwise process until the entire genome was reconstituted in yeast. Since next-generation sequence analysis revealed that the low-passage-number isolate represented a mixture of parental and fibroblast-adapted genomes, we selectively modified individual DNA fragments of fibroblast-adapted Toledo (Toledo-F) and again used TAR assembly to recreate parental Toledo (Toledo-P). Linear, full-length HCMV genomes were transfected into human fibroblasts to recover virus. Unlike Toledo-F, Toledo-P displayed characteristics of primary isolates, including broad cellular tropism *in vitro* and the ability to establish latency and reactivation in humanized mice. Our novel strategy thus enables *de novo* cloning of CMV genomes, more-efficient genome-wide engineering, and the generation of viral genomes that are partially or completely derived from synthetic DNA.

**IMPORTANCE** The genomes of large DNA viruses, such as human cytomegalovirus (HCMV), are difficult to manipulate using current genetic tools, and at this time, it is not possible to obtain, molecular clones of CMV without extensive tissue culture. To overcome these limitations, we used synthetic biology tools to capture genomic fragments from viral DNA and assemble full-length genomes in yeast. Using an early passage of the HCMV isolate Toledo containing a mixture of wild-type and tissue culture-adapted virus. we directly cloned the majority sequence and recreated the minority sequence by simultaneous modification of multiple genomic regions. Thus, our novel approach provides a paradigm to not only efficiently engineer HCMV and other large DNA viruses on a genome-wide scale but also facilitates the cloning and genetic manipulation of primary isolates and provides a pathway to generating entirely synthetic genomes.

## Abstract (precis)

The genomes of large DNA viruses, such as human cytomegalovirus (HCMV), are difficult to manipulate using current genetic tools, and at this time, it is not possible to obtain, molecular clones of CMV without extensive tissue culture. To overcome these limitations, we used synthetic biology tools to capture genomic fragments from viral DNA and assemble full-length genomes in yeast. Using an early passage of the HCMV isolate Toledo containing a mixture of wild-type and tissue culture-adapted virus. we directly cloned the majority sequence and recreated the minority sequence by simultaneous modification of multiple genomic regions. Thus, our novel approach provides a paradigm to not only efficiently engineer HCMV and other large DNA viruses on a genome-wide scale but also facilitates the cloning and genetic manipulation of primary isolates and provides a pathway to generating entirely synthetic genomes.

## INTRODUCTION

Persistent infection with human cytomegalovirus (HCMV) is widely prevalent in the human population and mostly asymptomatic in immunocompetent individuals (1). However, HCMV can cause disease upon immunosuppression, and HCMV is the most frequent infectious cause of congenital defects such as hearing loss (2). For this reason, vaccine development against HCMV has been given high priority, and multiple approaches have been and are being tried, so far with limited success (3). Due to its unique immunogenicity and T cell programming potential, recombinant CMV has also shown great promise as a vaccine vector for chronic infections such as HIV in nonhuman primate models, and clinical development of HCMV-based vectors is under way (4).

The development of vaccines against HCMV and vaccines based on HCMV, as well as virological and immunological studies *in vitro* and *in vivo*, require the genetic manipulation of CMV genomes. However, the large size of CMV genomes (>230 kb) renders genetics challenging. Initially, recombinant CMV was generated by marker rescue (5) or by *in vivo* recombination of overlapping cosmids (6). However, both techniques have been superseded by the cloning of CMV as a bacterial artificial chromosome (BAC) which allowed the genetic manipulation of complete CMV genomes in Escherichia coli (7). BAC clones are generated by replacing a genomic region that is nonessential for growth in tissue culture with a bacterial origin of replication together with a selectable marker using *in vivo* recombination in infected fibroblasts. Upon transformation of the BAC into E. coli, the deleted region can be reinserted, thus generating a full-length viral genome containing the BAC cassette. Recombinant virus is reconstituted upon transfection of the BAC into healthy diploid fibroblast cells followed by the recombinase-mediated excision of the BAC cassette that leaves a loxP “scar” in the genome (8).

Although BAC recombineering has revolutionized the genetic manipulation of large DNA virus genomes, several disadvantages remain. First and foremost, BAC cloning requires extensive virus passaging in tissue culture, since the insertion of the BAC cassette and subsequent selection of recombinants still use the traditional marker rescue method. This procedure prevents the direct cloning of HCMV genomic DNA isolated from clinical samples and rapidly selects for viral subpopulations and viral mutants adapted to growth in tissue culture (9). Since recent work revealed an astounding diversity of HCMV genomes in infected individuals (10), the requirement for tissue culture limits our ability to characterize individual clones within a swarm of genomes. As a result, relatively few HCMV genomes have been cloned as a BAC, and all of the original BAC clones show typical signs of tissue culture adaptation (11, 12). Furthermore, the restoration of the actual sequence present in the primary isolate often requires multiple sequential cloning steps (9).

Recent advances in the assembly and editing of large DNA fragments and entire bacterial genomes by synthetic biology methods enable microbial genome engineering at an unprecedented scale and scope (13). Several bacterial genomes have been cloned and maintained in Saccharomyces cerevisiae, and completely synthetic versions of entire chromosomes of several *Mycoplasma* species were assembled from DNA fragments (14, 15). These synthetic biology approaches thus take advantage of the unique ability of S. cerevisiae to capture and reliably join DNA fragments into entire heterologous chromosomes that can be successfully maintained as yeast artificial chromosomes (YAC). Particularly useful for direct cloning of DNA from a given sample is transformation-associated recombination (TAR) cloning in which linear DNA containing a yeast plasmid flanked by sequences homologous to a target sequence is transformed together with the target sequence (16). Upon recombination *in vivo*, the target sequence is then contained within a yeast plasmid.

Here, we explore the use of TAR cloning to directly clone genomic fragments of HCMV DNA preparations after limiting passaging in tissue culture followed by assembly of a full-length genome in Saccharomyces cerevisiae. As our test case, we used the earliest available passages of the HCMV isolate Toledo, the only low-passage-number isolate that has ever been used in experimental infection of humans. Toledo was isolated from a congenitally infected child, and the fourth passage was initially characterized in seropositive individuals (17) and later used to infect seronegative individuals or individuals who had been previously vaccinated with the serially passaged Towne strain (18). In both instances, there were clear immunological and clinical signs of infection as well as superinfection consistent with Toledo representing a fully infectious HCMV isolate representative of circulating strains. However, cloning and sequencing of the full-length genome of Toledo as a BAC revealed that, compared to other primary isolates, Toledo displayed genome inversions and deletions consistent with tissue culture adaptations (11). We now demonstrate that low-passage-number isolates of Toledo represented a mixture of both the fibroblast-adapted version and the parental sequence. Using TAR cloning, we generated both versions of Toledo and demonstrate that only the parental virus has the *in vitro* and *in vivo* hallmarks of a primary isolate.

## RESULTS

### Passage 7 of HCMV Toledo contains both wild-type and mutated ULb’ regions.

**FIG 1:**
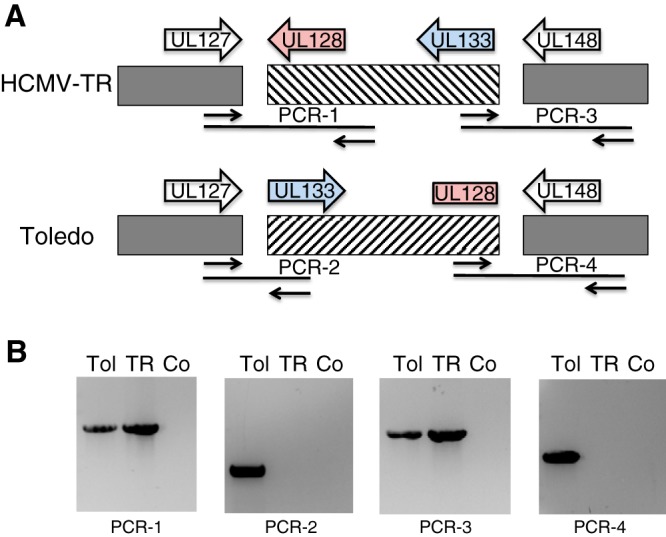
Passage 7 of HCMV Toledo contains a mixture of intact and inverted ULb’ regions. (A) Genomic organization of HCMV TR, representing a typical low-passage-number isolate, and HCMV Toledo, which shows an inversion of the UL133-UL128 region and a truncation of the UL128 ORF that removes the third exon and thus the terminal 71 amino acids. The approximate positions and directions of the primer pairs used for diagnostic PCR are also indicated. (B) PCR fragments obtained from P7 Toledo contain not only fragments expected for Toledo (Tol) (PCR-2 and PCR-4) but also fragments expected for a wild-type strain of HCMV (PCR-1 and PCR-3). Co, control.

Compared to other primary isolates, BAC-cloned HCMV Toledo displayed an inversion in the so-called ULb’ region that is frequently mutated upon multiple passages of HCMV. Specifically, BAC-cloned Toledo displayed an inversion resulting from a recombination event within sequences located between UL128 and UL129 at one end and UL133 and UL148a at the other end (Fig. 1A) (11). This recombination event led to a truncation of UL128 (19), a chemokine-like protein that is a subunit of the pentameric complex gH/gL/UL128/UL130/UL131A (20, 21). While the pentameric complex promotes infection of endothelial, epithelial, and myeloid cells, mutations in the nonessential subunits UL128, UL130, and UL131A are selected for upon passaging in fibroblasts, presumably due to the fact that pentamer-deficient viruses are less cell associated (12). Since pentameric complex mutants of nonhuman primate CMVs are attenuated *in vivo* (22), this fibroblast adaptation was at odds with the clinical observations in volunteers inoculated with the Toledo isolate. Therefore, we determined whether early passages of Toledo already contained this deletion by isolating DNA from supernatants of fibroblasts infected with Toledo passage 7 (P7), the earliest passage still available, and performing diagnostic PCR of genes adjacent to UL128 of UL133 and the native versus inverted configuration. In the HCMV isolate TR, used as a control, UL128 is adjacent to UL127, whereas UL133 is adjacent to UL148 as indicated by the appropriate PCR fragments PCR-1 and PCR-3 (Fig. 1B). Interestingly, the same fragments were also amplified from P7 Toledo, indicating a noninverted genome orientation similar to that of TR. In addition, P7 Toledo also contained genomes in the inverted configuration in which UL128 is adjacent to UL148 (PCR-4) and UL133 is adjacent to UL127 (PCR-2) (Fig. 1B). Thus, the early passages of Toledo used in clinical trials consisted of a mixture of wild-type and fibroblast-adapted genome configurations.

### Next-generation sequence analysis of an early passage of HCMV Toledo.

**FIG 2:**
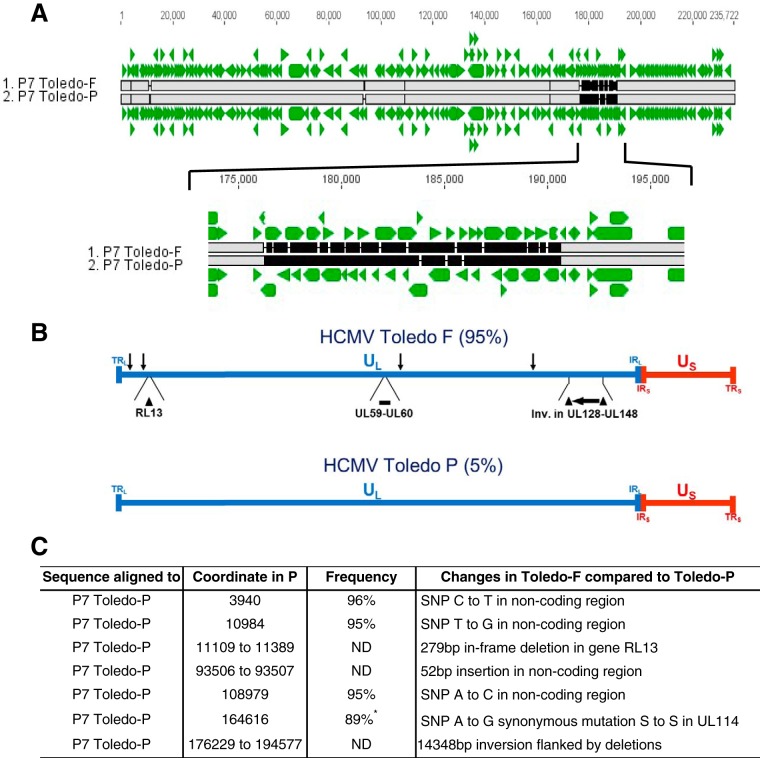
Sequence comparison between the fibroblast-adapted majority sequence (Toledo-F) and the parental minority sequence (Toledo-P) in passage 7 of HCMV Toledo. (A) Genome alignment of HCMV Toledo-F with the parental Toledo-P. The green arrowheads indicate the HCMV ORFs. The gray bars indicate sections of agreement, the black lines indicate differences, and the black bars indicate deletions and insertions. (B) Locations of the inverted UL133-UL128 (UL133-128) region (black horizontal arrow) flanked by deletions (black triangles), the deletion of 279 bp in RL13 (black triangle), and the insertion of 52 bp in the UL59-UL60 region (black bar), as well as the locations of SNPs (black vertical arrows) within the P7 Toledo-F genomic sequence. The ratio of P7 Toledo-F to P7 Toledo-P was approximately 95% to 5%, respectively. The positions of long and short terminal repeats (TR_L_ and TR_S_, respectively) and long and short inverted repeats (IR_L_ and IR_S_, respectively) are shown. (C) List and frequency of nucleotide differences in the minority sequence Toledo-P compared to Toledo-F. For SNP 164616, the coverage of reads was at least twofold lower than the other loci (indicated by the asterisk), rendering the estimation of frequency less accurate. P, parent; ND, not determined.

To further examine the genome content of low-passage-number Toledo samples in an unbiased fashion, we employed Illumina HiSeq paired-end sequencing on viral DNA isolated from the supernatant of fibroblasts infected with P7 Toledo. The depth of sequence coverage was sufficient to obtain the full-length sequences of both the majority sequence representing the fibroblast-adapted Toledo strain and the minority, presumably parental, wild-type sequence. We will refer to the fibroblast-adapted Toledo strain and the presumably parental wild-type strain as Toledo-F and Toledo-P, respectively. Toledo-F was mostly identical to the Toledo sequence deposited into GenBank (GenBank accession number GU937742.2). However, a few single nucleotide polymorphisms (SNPs) were identified, most of them synonymous mutations (see Fig. S1 in the supplemental material). These SNPs are either due to sequencing errors in the GenBank sequence or mutations that occurred during further passaging and cloning of the Toledo isolate. In order to model the sequence of the Toledo-P genome and the Toledo-F genome, we edited the reference genome sequence (GenBank accession number GU937742.2) based on our sequencing results. Individual SNPs were allocated to either one or both Toledo genomes based on the following criteria. If the variation was homogeneous, it was applied to both genomes. If the variation was heterogenous, the major allele was applied to the fibroblast-adapted genome, while the minor allele was applied to the parental genome. Usually, for heterogeneous variations, one of the alleles would match the allele in the reference genome, such that “applying” it would be to take no action to the derivative genome at that locus. Alignment of the full-length sequences of Toledo-F and Toledo-P revealed that, in addition to the inversion of the UL128-UL133 fragment which is flanked by two small deletions, Toledo-F had a 279-bp in-frame deletion in the RL13 gene, as previously reported (23), a 59-bp insertion in the intergenic region between UL59 and UL60, as well as several SNPs in the UL region (Fig. 2A and B). By comparing the sequence coverage (number of reads) of Toledo-F versus Toledo-P at the SNP loci, we were able to estimate the ratio of the two sequences in the viral DNA preparation. The P7 Toledo-F genomes represented the vast majority of sequences, whereas only 5% of the reads belonged to Toledo-P (Fig. 2C). Since only these two genome sequences were detected in the P7 sample, it is highly likely that P7 represents an intermediate mixture in which most of the culture has been taken over by a faster-growing, fibroblast-adapted variant, with some of the original isolate remaining. Similar observations have been reported upon serial passaging of the primary isolate Merlin where mutations in RL13 occurred within three passages, and mutations in the UL128-to-UL131A (UL128-131A) region upon subsequent passages (12). Since clinical studies were performed with passage 4 Toledo, these data also suggest that the percentage of non-fibroblast-adapted, wild-type, sequence was likely higher, although no passage 4 samples remain, making it impossible to determine the exact composition.

10.1128/mSphereDirect.00331-17.1 FIG S1 Sequence comparison between HCMV Toledo (GenBank accession number GU937742.2) and the majority sequence in P7 HCMV Toledo (P7 Toledo-F). (A) Genome alignment of the NGS results obtained by sequencing P7 HCMV Toledo with HCMV Toledo (GenBank accession number GU937742.2). The green bar at the top indicates the percentage of nucleotide identity between both virus sequences with green being 100% identical and yellow being less than 100% identity. The gray bars indicate sections of agreement, the black lines indicate differences, and the black bars indicate deletions and insertions. The green arrows indicate the HCMV ORFs. (B) List of nucleotide differences between the published sequence and P7 Toledo-F. The SNP in the homopolymeric sequences is possibly a false-positive result. Download FIG S1, PDF file, 0.2 MB. Copyright © 2017 Vashee et al. 2017 Vashee et al. This content is distributed under the terms of the Creative Commons Attribution 4.0 International license.

### TAR cloning of HCMV Toledo.

**FIG 3:**
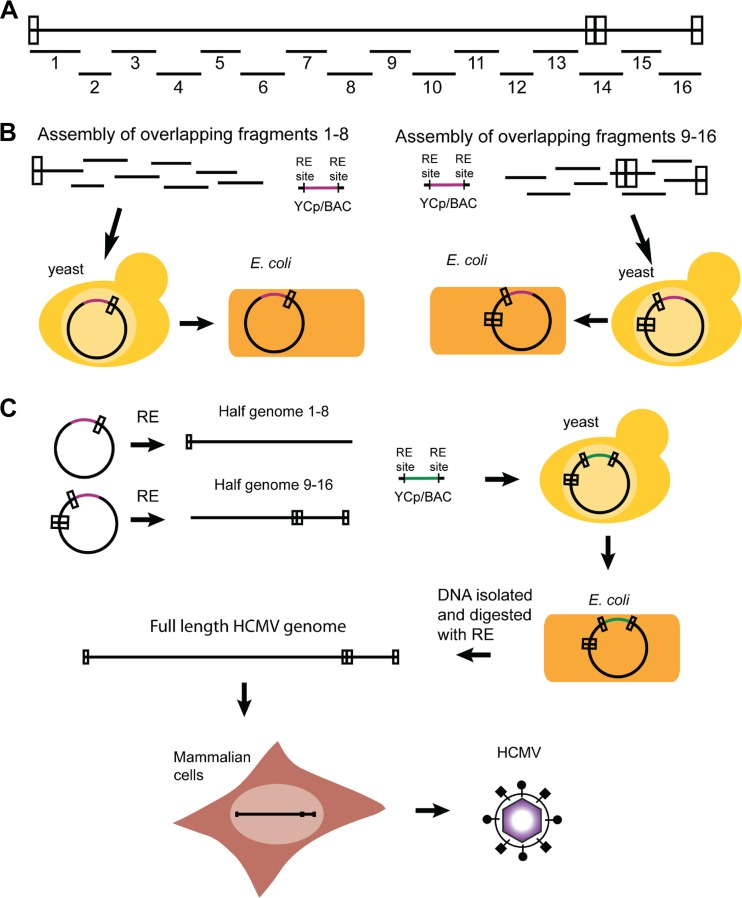
Assembly of full-length HCMV genomes from overlapping genomic fragments. (A) Sixteen genomic fragments of approximately 15 kbp each span the entire sequence of the HCMV genome and overlap the adjacent fragment(s) by at least 76 bp. (B) Half genomes of HCMV were assembled separately from fragments 1 to 8 and fragments 9 to 16. The corresponding fragments were pooled with a linear YCp/BAC vector that harbored terminal homology to each end of the half genome and flanked by a unique restriction enzyme (RE) site. These DNAs were transformed into S. cerevisiae (yeast) cells, and transformants were screened by PCR for assembly of the half genomes. The DNA from positive transformants was transformed into E. coli cells to produce high concentration DNA stocks. (C) Plasmid DNAs of both half genomes were isolated and digested to linearize the HCMV DNAs. The half genomes were transformed into yeast cells with a linear YCp/BAC vector, which has homology to the terminal repeats of HCMV flanked by unique restriction sites and has a different marker for selection in yeast than was used for the half genome assemblies. DNA from positive transformants was isolated and transformed into E. coli. DNA was isolated from E. coli, and the viral genome was released by digestion with a restriction enzyme targeting flanking sequences. Transfection of the DNA into mammalian cells reconstituted live HCMV.

**FIG 4:**
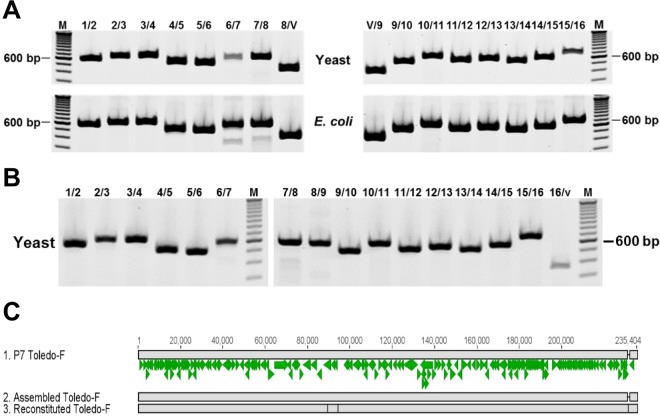
Assembly of Toledo-F genome. (A) Junction PCR analysis of assembled half genomes. Representative agarose gels after PCR amplification showing the presence of appropriate junctions between the Toledo-F fragments (1, 2, etc.) for the half genomes, fragments 1 to 8 (left panels) and fragments 9 to 16 (right panels) in yeast and E. coli. For the lane headings, numbers give the Toledo-F fragment numbers and V refers to vector for the junctions tested. The positions of molecular size markers in the M lanes are given to the left of right of the gels. (B) Junction PCR analysis of assembled full-length genome. Representative agarose gels after PCR amplification showing the presence of all the appropriate junctions between the Toledo-F fragments (1, 2, etc.). For the lane headings, numbers give the Toledo-F fragment numbers and v refers to vector for the junctions tested. (C) Sequence comparison between HCMV P7 Toledo-F, assembled Toledo-F, and reconstituted Toledo-F virus. The genome alignment is shown as described in the legend to Fig. 2. Four SNPs were identified in the reconstituted Toledo-F compared to assembled or P7 Toledo-F at positions 89516 (C to C), 94187 (C to T), 94226 (G to T), and 231238 (G inserted). However, all of these SNPs were in regions of lower sequencing coverage and are therefore more likely to be sequencing errors than true mutations.

The observed presence of different variants within a given primary isolate illustrates a typical challenge for the cloning of HCMV genomes. Continued passage of Toledo likely resulted in the presence of only the Toledo-F population so that two independently derived BAC clones correspond to Toledo-F (11, 24). Moreover, four different chimeras of HCMV Toledo cloned as overlapping cosmids with the vaccine strain HCMV Towne were exclusively based on Toledo-F which might explain their limited infectivity and immunogenicity in clinical studies (25–27). To enable the cloning of primary isolates with limited propagation in tissue culture, we therefore used the P7 Toledo sample as a test case for a new TAR cloning-based strategy applicable to HCMV isolates in general (Fig. 3). By aligning a number of known HCMV isolates, we identified highly conserved sequences that were used to design 40-bp-long, synthetic “TAR hooks” (Table S1) for the cloning of the entire genome as 16 overlapping fragments (Table S2). Pairs of adjacent TAR hooks flanking 10- to 20-kb regions were inserted into a yeast centromeric plasmid with BAC sequence (YCpBAC) and transformed into yeast together with sheared P7 HCMV Toledo DNA (Fig. S2A). Each of the 16 fragments which contain 80 bp of homology between adjacent fragments as well as flanking I-SceI sites was characterized by PCR and restriction analysis as shown for fragment TAR04 (Fig. S2B to D). The efficiency for cloning the HCMV fragments ranged from 20% (TAR15) to 80% (TAR03). Since multiple attempts to obtain full-length genomes from the 16 overlapping DNA fragments failed, we assembled the full-length HCMV Toledo in two steps. In the first step, we generated two half genomes by cotransforming overlapping DNA fragments 1 to 8 and fragments 9 to 16 together with the YCpBAC vector into S. cerevisiae (Fig. 4A). The resulting half genomes were also transferred into E. coli and characterized by PCR analysis (Fig. 4A). Assembly of the half genomes was very efficient. Positive results were observed in 95% of the clones that were screened. To generate a full-length genome, the half genomes were released from the YCpBAC by I-SceI or PacI restriction digestion and transformed together with linearized YCpBAC containing TAR hooks for the beginning and end of the HCMV genome (Table S1). The resulting full-length genomes in S. cerevisiae were analyzed by junction PCR analysis (Fig. 4B), and positive clones were transformed into E. coli. The success rate for assembling full-length Toledo-F genomes in yeast was approximately 15%, and the transfer of the full-length genomes to E. coli was close to 100% efficient. Next-generation sequence analysis showed that the TAR-assembled HCMV genome was identical to the P7 Toledo-F sequence present in the low-passage-number sample (Fig. 4C).

10.1128/mSphereDirect.00331-17.2 FIG S2 TAR cloning of overlapping genomic fragments of HCMV Toledo. (A) Depiction of cloning TAR fragments. A linear TAR cloning vector containing YCp and BAC sequences with 40 bp of homology to the desired fragment of HCMV (in this example, TAR fragment 4) and I-SceI restriction sites was cotransformed with sheared P7 HCMV DNA into yeast cells. Yeast transformants were screened by PCR, and positive clone DNA was transferred to E. coli. (B) TAR cloning and analysis of HCMV fragment 4 as an example. (Left) Generation of linear TAR cloning vector. To clone HCMV fragment 4, a linear TAR cloning vector was amplified by PCR using pCC1BAC-his3 as the template and Con04F and Con04R (Table S1) as primers. (C) Junction analysis by PCR. Yeast transformants of HCMV TAR clones were screened by PCR using detection primers, Det04F and RCO493 (Table S1) for the forward junction and Det04R and RCO495 (Table S1) for the reverse junction. (D) Restriction enzyme analysis of a positive HCMV fragment 4 clone. DNA from a positive yeast clone was isolated and transformed into E. coli. Plasmid DNA from E. coli was isolated and incubated with the designated REs and then analyzed by agarose gel electrophoresis. Expected DNA band sizes were observed. Blue diamonds indicate vector DNA bands, and red circles indicate fragment 4 DNA bands that comigrate with the vector bands. Download FIG S2, PDF file, 0.04 MB. Copyright © 2017 Vashee et al. 2017 Vashee et al. This content is distributed under the terms of the Creative Commons Attribution 4.0 International license.

10.1128/mSphereDirect.00331-17.3 TABLE S1 Primers used in this study. Download TABLE S1, DOCX file, 0.02 MB. Copyright © 2017 Vashee et al. 2017 Vashee et al. This content is distributed under the terms of the Creative Commons Attribution 4.0 International license.

10.1128/mSphereDirect.00331-17.4 TABLE S2 Design of HCMV fragments. Download TABLE S2, DOCX file, 0.02 MB. Copyright © 2017 Vashee et al. 2017 Vashee et al. This content is distributed under the terms of the Creative Commons Attribution 4.0 International license.

To assemble the Toledo-P genome also, we took advantage of the ability to simultaneously change the sequence of each of the 16 fragments either by mutagenesis or by gene synthesis. The major differences between Toledo-P and Toledo-F are located on three fragments: fragment TAR01 contains the RL13 sequence, fragment TAR07 contains open reading frames (ORFs) 59 and 60, and fragment TAR13 contains the UL128-UL133 (UL128-133) region. There were also four SNP differences between Toledo-P and Toledo-F (Fig. 2). However, since these either occurred in noncoding regions or were synonymous, we did not modify them in our assembly of Toledo-P.

**FIG 5:**
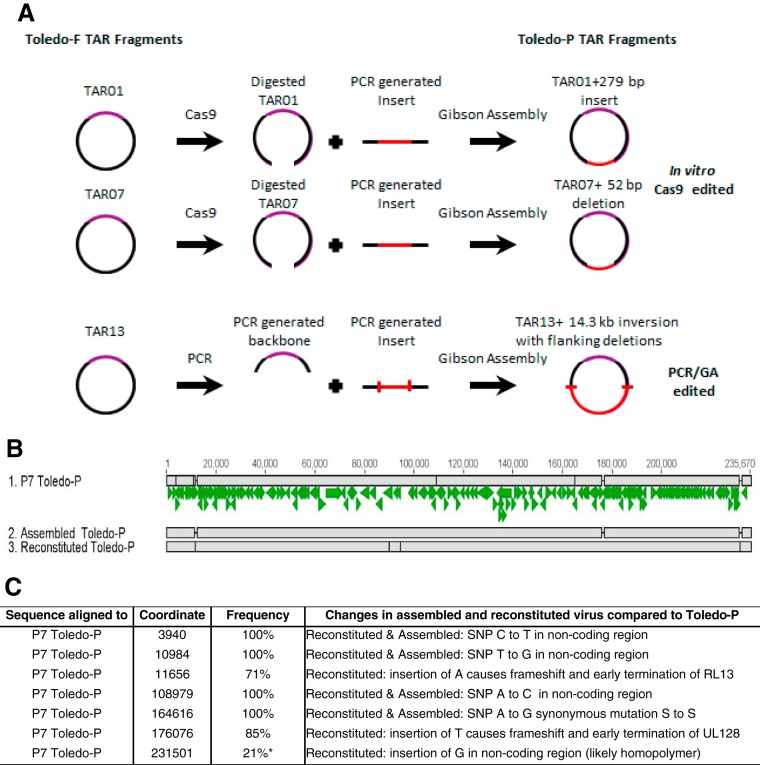
Modification of fragments TAR01, TAR07, and TAR13 and generation of full-length Toledo-P. (A) Generating Toledo-P fragments TAR01, TAR07, and TAR13. A 279-bp sequence was added to RL13 (TAR01), a 52-bp sequence was deleted from the intergenic region of UL59-UL60 (TAR07), and the inversion (TAR13) was repaired. GA, Gibson Assembly. (B) Sequence comparison between HCMV P7 Toledo-P, assembled Toledo-P, and reconstituted virus. The genome alignment is shown as described in the legend to Fig. 2 for the sequence of Toledo-P cloned into YCpBAC upon repair of TAR01, TAR07, and TAR13 of Toledo-F (assembled Toledo-P) and the sequence of virus after 4 passages *in vitro* (reconstituted Toledo-P). (C) List and frequency of nucleotide differences in the assembled and reconstituted Toledo-P compared to the minority sequence Toledo-P present in P7 Toledo. The locations, frequencies, and sequences of these changes in the reconstituted virus are shown compared to P7 Toledo-P. Assembled Toledo contained four SNPs compared to the P7 Toledo-P sequence, since these SNPs were not modified in TAR-cloned Toledo-F. The four SNPs were also found in reconstituted Toledo-P. In addition, reconstituted Toledo-P contained three SNPs in the indicated fraction of the genome. The SNPs in RL13 and UL128 were found in the majority of sequences. However, SNP 231501 (indicated by the asterisk) is contained in a homopolymeric stretch of G’s and might be a false-positive result due to a misalignment of the sequencing reads. Additional SNPs were identified in the reconstituted Toledo-P at positions 89795 (C to G), 94414 (C to T), and 94453 (G to T). However, all of these SNPs were in regions of lower sequencing coverage and are therefore more likely to be sequencing errors than true mutations.

TAR01 and TAR07 corresponding to the Toledo-P sequence were generated by *in vitro* Cas9 digestion followed by assembly with a PCR fragment containing the Toledo-P sequence (Fig. 5A). TAR13 was modified to contain the appropriate Toledo-P sequence by first amplifying the inverted region with primers that not only contained the additional sequences for Toledo-P but also contained sequences to restore the proper orientation of the UL128-133 region. A YCpBAC with the remaining Toledo sequences was also amplified, and the two fragments were assembled utilizing Gibson Assembly (SGI-DNA) and transformation into E. coli.

The resulting TAR plasmids were sequenced to confirm that the respective Toledo-P fragments had been generated (data not shown). The full-length Toledo-P genome was then generated from two half genomes in which Toledo-P fragments TAR01, TAR07, and TAR13 replaced the corresponding Toledo-F fragments. The assembly of the full-length Toledo-P genome in yeast and transfer to E. coli were not as robust as those of Toledo-F. Only 3% of the yeast Toledo-P clones were correct (compared to 15% for Toledo-F), and the efficiency of Toledo-P full-length genome transfer to E. coli was 10% (compared to almost 100% for Toledo-P). The reasons for this relative inefficiency for Toledo-P are not yet understood. The resulting full-length YCpBAC containing Toledo-P genome was analyzed by next-generation sequencing (NGS) (Fig. 5B and C). Except for the four SNPs that were not repaired (and hence occur in 100% of the assembled virus), the sequence of the assembled full-length Toledo-P YCpBAC corresponded to the minority, Toledo-P sequence in the P7 Toledo sample.

### Reconstitution and characterization of TAR-cloned HCMV Toledo.

**FIG 6:**
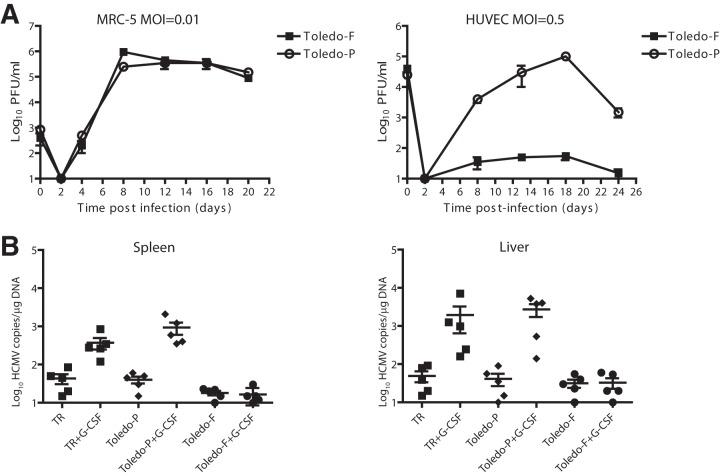
*In vitro* and *in vivo* comparison of cloned HCMV Toledo-P and Toledo-F. (A) *In vitro* growth of HCMV Toledo-P and Toledo-F. (Left) Multistep growth curves in MRC-5 cells. A total of 80,000 cells per well in a 24-well plate were infected in duplicate at an MOI of 0.01. After 90 min, the inoculum was removed, the cells were washed three times with PBS, and fresh DMEM plus 10% FBS was added. Supernatants from infected cells (500 µl) were harvested at the indicated times postinfection. (Right) Growth curves in endothelial cells (HUVECs). A total of 80,000 cells per well in a 24-well plate were infected in duplicate at am MOI of 0.5. After 90 min, the inoculum was removed, the cells were washed three times with PBS and fresh EGM plus 2% FBS was added. Supernatants were harvested at the indicated times postinfection. All the samples were titrated by plaque assay on MRC-5 cells. (B) Infection of humanized NSG (hNSG) mice with HCMV TR, Toledo-P, or Toledo-F. Humanized mice (12 to 14 weeks after engraftment) were infected via intraperitoneal (i.p.) injection using human fibroblasts previously infected with HCMV TR, Toledo-P, or Toledo-F at approximately 5 × 10^5^ PFU per mouse. A control group of engrafted mice were mock infected using uninfected fibroblasts (data not shown). At 8 weeks postinfection, mice were split into two groups, and one group was treated with 100 µl of G-CSF (300 mg/ml) using a subcutaneous micro-osmotic pump and 125 μg AMD3100 administered i.p. to mobilize hematopoietic progenitor cells (HPCs). One week after mobilization of the HPCs, the mice were sacrificed, and their spleens and livers were harvested. HCMV genomes were analyzed using quantitative PCR performed on 1 µg of total DNA and using an HCMV UL141 probe. The increased recovery of viral genomes was statistically significant for both TR and Toledo-P in liver and spleen (liver, *P* = 0.03 for TR and *P* = 0.02 for Toledo-P; spleen, *P* = 0.002 for TR and *P* = 0.005 for Toledo-P).

The TAR-cloned HCMV Toledo genomes were released from the YCpBAC by restriction enzyme digestion targeting the flanking restriction sites with PacI endonuclease. To recover virus, the released full-length genomes were transfected into MRC-5 fibroblasts. After 4 passages in MRC-5 cells, purified viral stocks were generated for *in vitro* and *in vivo* characterization. At the same time, viral DNA was extracted by the Hirt method and sequenced by NGS. Comparison of the Toledo-P virus with the YCpBAC sequence revealed frameshift mutations in RL13 amino acid residue 193 and in UL128 amino acid residue 154 (Fig. 5B and C). The frameshift mutation in RL13 and UL128 were found in approximately 71% and 85% of the genomes, respectively, suggesting that mutations in these gene regions are rapidly selected for even when starting with a molecular clone. However, while the RL13 mutation truncates the protein by 50% and likely inactivates the protein, the frameshift in UL128 eliminates only the final 16 amino acids. Since UL128 is a subunit of the pentameric complex that facilitates entry into nonfibroblast cells, we determined whether the UL128 truncation would affect the anticipated ability of Toledo-P to infect endothelial cells. In a multistep growth curve (multiplicity of infection [MOI] of 0.01) in MRC-5 cells, both Toledo-P and Toledo-F generated comparable titers of virus in the supernatant and with comparable kinetics (Fig. 6A). In contrast, only Toledo-P was able to grow in human umbilical cord endothelial cells (HUVECs), whereas Toledo-F did not grow (Fig. 6A). These data suggest that Toledo-P retained a functional pentameric complex despite the truncation in UL128.

We previously reported a humanized mouse model in which primary isolates of HCMV are able to establish latency and can be reactivated upon mobilization of myeloid cells from the bone marrow (28–30). In contrast, laboratory-adapted strains such as AD 169 are unable to do so (P. Caposio, unpublished observations). To compare Toledo-P and Toledo-F in this model, we engrafted NOD-scidIL2Rγc null (NSG) mice with CD34^+^ human progenitor cells. At 12 to 14 weeks after engraftment, mice were infected via intraperitoneal injection of human fibroblasts infected with HCMV TR, HCMV Toledo-P, or HCMV Toledo-F. At 8 weeks postinfection, the mice were split into two groups, and one group was treated with granulocyte colony-stimulating factor (G-CSF) and AMD3100 (see Materials and Methods) was administered intraperitoneally to mobilize human monocytes/macrophages. At 1 week after mobilization, the mice were sacrificed. Total DNA was extracted from the spleens and livers, and HCMV genomes were analyzed using quantitative PCR. When comparing G-CSF-treated to untreated groups, we were able to detect quantitative differences in viral DNA loads in the spleen and liver for both HCMV TR and Toledo-P (Fig. 6B). In contrast, no increase in viral genome copies were observed for Toledo-F. Moreover, compared to HCMV-TR and Toledo-P, genome copy numbers tended to be lower even in nonmobilized mice infected with Toledo-F. Thus, Toledo-P and Toledo-F strongly differ in their ability to establish latency and reactivate *in vivo*. Only Toledo-P behaves similarly to other primary isolates (TR, TB40E) in this model, whereas Toledo-F resembles laboratory-adapted strains like AD 169.

## DISCUSSION

By applying synthetic biology tools, specifically TAR cloning, we were able to generate a molecular clone of HCMV, the largest human herpesvirus, directly from DNA extracted from a primary isolate after limited passaging *in vitro*. Thus, this method potentially enables the direct cloning of CMV from clinical samples, e.g., from viral DNA isolated from congenitally infected infants, without the need for tissue culture. The “TAR hooks” used to generate the 16 genome fragments were designed to represent highly conserved genome sequences so that most HCMV genomes can be cloned by this method. However, in case of sequence mismatches, TAR hooks can be easily modified according to sequencing results obtained from the same DNA sample. This cloning method can also be applied to new DNA virus isolates for which *in vitro* culture conditions have not been established (31). Probably due to the large size of the HCMV genome (>230 kb) and the presence of terminal repeats at each end of the genome, we were unable to recover a full-length genome by cotransformation of all 16 10- to 20-kb fragments. Instead, we generated two half genomes of eight fragments each, which were combined into a full-length genome in a second recombination step. In contrast, smaller herpesvirus genomes like herpes simplex virus 1 (HSV-1) can be assembled in one step (43). TAR cloning is thus the first method that permits direct cloning of herpesviral genomes. In addition, by synthesizing individual TAR fragments, this method can be used to generate partially or fully synthetic HCMV genomes. Synthetic viral genomes have been successfully assembled from individual fragments for smaller viruses, e.g., a 31-kb consensus severe acute respiratory syndrome (SARS) coronavirus genome from bats (32). However, to date, this has not been possible for large DNA viruses due to the lack of a suitable technology. As the parallel chemical synthesis of large DNA fragments is becoming feasible and affordable (33), the assembly of synthetic HCMV genomes from individual fragments in yeast described here will ultimately enable construction of a completely synthetic genome.

By modifying the sequence of any of the 16 fragments independently, it is also possible to simultaneously change multiple genomic loci as exemplified here by the reconstruction of the Toledo-P sequence by exchanging three fragments of Toledo-F. Thus, various full-length genomes that are ready to be transfected can be assembled from the fragments in about 3 to 4 weeks under ideal conditions. In contrast, the introduction of multiple mutations by BAC technology requires multiple steps of sequential mutagenesis. Moreover, the final assembled HCMV genome does not contain any heterologous sequences once released by restriction digestion, whereas removal of the BAC cassette by Cre recombinase leaves a loxP site in the viral genome. Due to this versatility, TAR-based cloning will also enable the generation of hybrid genomes, e.g., between different cytomegalovirus (CMV) strains or species to facilitate mapping of strain- or species-specific traits. The fact that native viral DNA that does not contain residual bacterial sequences is excised from the YCpBAC prior to transfection will additionally facilitate the recovery of live virus without the need of further genome manipulations such as Cre-based excision of the BAC cassette.

As a test case for direct cloning, we used the low-passage-number isolate Toledo, a storied virus derived from a congenitally infected boy in Toledo, Ohio. Passage four of HCMV Toledo was used in human challenge studies, and these clinical trials remain the only examples for experimental infection of humans with low-passage-number HCMV (17). It was observed that both HCMV Towne-vaccinated individuals as well as naturally seropositive individuals showed clinical symptoms such as mild mononucleosis-like syndrome when challenged with >1,000 PFU of P4 Toledo, whereas seronegative individuals developed symptoms with doses as low as 10 PFU (18). However, in light of our deep sequencing analysis showing that the Toledo isolate is a mixture of intact Toledo-P and mutated Toledo-F, it is likely that these symptoms were caused by the intact fraction of the inoculum, suggesting that the actual PFU needed to overcome anti-CMV immunity might have been even lower.

A significant difference between Toledo-P and Toledo-F in their ability to establish persistent infection *in vivo* is supported by our experiments in humanized mice. Toledo-F was barely able to establish latency in this model, and we did not observe an increase in the viral genome copy number upon mobilization of monocytes/macrophages, an indicator of reactivation and dissemination. In contrast, similar to other primary isolates, Toledo-P established robust latency and reactivation upon monocyte/macrophage mobilization. This ability correlates to some extent with the broader cell tropism displayed by pentamer-intact HCMV. However, it is also possible that the inversion of the ULb’ fragment affects gene expression within the UL133-138 gene locus which has been implicated in viral latency as well as infection of endothelial cells (29). Thus, multiple mutations could contribute to the attenuated phenotype of Toledo-F *in vivo*.

Interestingly, Toledo-P rapidly adapted to tissue culture conditions by mutating RL13 and UL128, two of the gene regions that were mutated in Toledo-F and that were repaired in the Toledo-P YCpBAC. Thus, even when HCMV Toledo-P is recovered from a molecular clone that was intact, mutations in these genes are rapidly selected for *in vitro*. Thus, it seems likely that the Toledo-F variant was not present within the initial isolate but resulted from random mutation and selection upon passaging *in vitro*. The rapid selection for mutations in RL13 and the nonessential pentamer subunits UL128, UL130, and UL131A from an intact molecular clone have been very well documented for the low-passage-number isolate Merlin (9). This work demonstrated that intact RL13, a viral Fc receptor (34, 35), limits viral propagation in all cell types, and mutants are selected upon very few passages (12). Thus, unless RL13 is conditionally expressed, RL13 mutants will arise *in vitro*. In contrast, UL128-131A mutants arise only upon passaging in fibroblasts (36) so the selection of these types of mutants can be avoided by growing virus in nonfibroblast cells such as epithelial cells. To maintain the genetic integrity of the repairs of RL13 and UL128 in Toledo-P, it would thus be required to control RL13 and UL128 expression via a conditional promoter as demonstrated for Merlin (37). However, despite the accumulation of mutations in both RL13 and UL128 in the majority of the viral genomes at passage 4, there was a clear difference in the ability of Toledo-P to infect endothelial cells and to infect monocyte/macrophages in NSG mice. One possible explanation is that these differences were due to the residual intact virus, similar to the situation in the passage 4 isolate used in clinical trials. Alternatively, the truncation in UL128 of P4 Toledo-P might still retain some pentamer function, a possibility that is supported by mutational studies that demonstrated a limited impact of C-terminal mutations of UL128 on endothelial cell infection (38). Clonal separation of the intact versus mutated version of Toledo will be required to distinguish between these possibilities.

Taken together, our data thus suggest that due to multiple mutations, Toledo-F is severely limited in its ability to establish and maintain latent infection. Importantly, cosmid clones of Toledo-F were previously used to generate chimeric viruses with the Towne vaccine strain in order to generate a vaccine strain that is less attenuated than the highly passaged Towne strain (25, 26). However, all four different chimeric viruses were unable to elicit clinical or immunological symptoms in HCMV-seropositive individuals at doses up to 1,000 PFU (26). Moreover, although CD8^+^ T cell responses were detected in seronegative individuals inoculated with two of the chimeras, the CD8^+^ T cell responses were weak (27) and did not demonstrate the typical expansion of effector memory T cells observed with CMV infection (25). The finding that Towne/Toledo-F chimeras were unable to recapitulate the immune response naturally elicited by HCMV correlates with the lack of Toledo-F to reactivate upon monocyte mobilization, suggesting that the chimeric viruses were likely unable to establish the persistent infection required to elicit and maintain effector memory. By modifying individual TAR fragments in Toledo-P, it is now possible to design a new generation of HCMV Toledo-based vaccines and vaccine vectors with the goal of retaining the ability of HCMV to elicit lasting effector memory while introducing safety features that eliminate viral pathogenesis.

## MATERIALS AND METHODS

### Cells and virus preparation.

Human primary embryonic lung fibroblasts (MRC-5; purchased from ATCC, Manassas, VA) were cultured in Dulbecco’s modified Eagle’s medium (DMEM) and supplemented with 10% fetal bovine serum (FBS), 10 mM HEPES, 1 mM sodium pyruvate, 2 mM l-alanyl-glutamine, 0.1 mM nonessential amino acids, 100 U/ml penicillin, and 100 µg/ml streptomycin. Human umbilical vein endothelial cells (HUVECs) were purchased from Lonza (cc-2519) and cultured in endothelial growth medium (EGM-2; Clonetics) containing 2% FBS, human recombinant vascular endothelial growth factor (VEGF), basic fibroblast growth factor (bFGF), human epidermal growth factor (hEGF), insulin-like growth factor 1 (IGF-1), hydrocortisone, ascorbic acid, heparin, gentamicin, and amphotericin B (1 μg/ml each) (high-serum medium). HCMV isolate TR was derived originally from a patient with AIDS-related HCMV retinitis (39). HCMV Toledo-P and Toledo-F viral DNA was extracted from E. coli using a Qiagen Midi-kit, digested with PacI, and electroporated into MRC-5 cells. Infectious virus was produced, and the titers of the virus were determined by plaque assay on MRC-5 cells as previously described (40).

### Plasmids.

Plasmids pCC1BAC-ura3 and pCC1BAC-his3 have been previously described (41).

### PCR for viral DNA.

Supernatant from fibroblasts infected with Toledo isolate passage 7 (P7) was used to extract viral DNA using the QIAamp MinElute virus spin kit (Qiagen). HCMV TR DNA was extracted with the same kit as a positive control. Two microliters of viral DNA was amplified with *Taq* 5× Master Mix (New England BioLabs [NEB]) using the following primers (indicated in the parentheses) for the regions and diagnostic PCR indicated: UL127 (5′-ATGTGCCAGCTTGATGTCGC-3′) and UL130 (5′-CGCCAAGATTTTTGGAGCGCAC-3′) (PCR-1); UL127 (5′-ATGTGCCAGCTTGATGTCGC-3′) and UL133 (5′-GGTTGTGAACTCACCGTCGG-3′) (PCR-2); UL133 (5′-GGTTGTGAACTCACCGTCGG-3′) and UL148 (5′-CGAGGCAGAACATCTCAACC-3′) (PCR-3); UL128 (5′-GAGGGCCTTACAGCCTATGG-3′) and UL148 (5′-CGAGGCAGAACATCTCAACC-3′) (PCR-4). The thermocycler parameters were 95°C for 2 min, followed by 30 cycles, with 1 cycle consisting of 94°C for 30 s, 55°C for 30 s, and 72°C for 1 min.

### HCMV Toledo genome sequencing and analysis.

Viral DNA was isolated from the supernatant of fibroblasts infected with P7 Toledo as described above, and 210 μl of isolated DNA was incubated with 30 μl of shrimp DNase to remove host DNA (2 U/μl) (Affymetrix) for 1 h at room temperature. Then, the shrimp DNase was inactivated by incubating at 70°C for 20 min. A 10-μl sample was removed for quantitative PCR (qPCR) analysis. Viral DNA was then purified from the larger remaining sample with the QIAamp MinElute virus spin kit (Qiagen) and eluted in 50 μl of buffer. qPCR was used to determine the removal of host DNA. Viral DNA was then used for sequencing using Illumina HiSeq paired-end sequencing. The sequence reads were sorted by barcode, trimmed, and mapped to HCMV Toledo (GenBank accession number GU937742.2) using CLC Bio’s (Qiagen) clc_ref_assemble_long program, and variations were then extracted and reported using CLC Bio’s find_variations program. Reported variations were manually inspected using CLC Bio’s assembly_viewer program, and variations lacking sufficient evidence (lack of spanning reads or insufficient high-quality coverage) were discarded. In some cases, large structural variations were analyzed and reported manually, for example, a large inversion with indels at the termini that occurred at low frequency had to be determined by bespoke bioinformatic methods to partition sequencing reads based on the presence or absence of junction sequences, and then reassembling the partitions of reads separately and mapping contigs back to the reference sequence.

The P7 Toledo-F, P7 Toledo-P, Assembled Toledo-F, Reconstituted Toledo-F, Assembled Toledo-P, and Reconstituted Toledo-P were aligned in Geneious (Biomatters Ltd., Auckland, New Zealand) using the PhyML package to the reference strain (HCMV Toledo, GenBank accession number GU937742.2) available from NCBI and to each other for identification of insertions, deletions, and single nucleotide polymorphisms (SNPs).

### Yeast transformation by spheroplast formation.

The S. cerevisiae strain VL6-48N (14) (MATα *his3*-Δ*200 trp1*-Δ*1 ura3*-Δ*1 lys2 ade2-101 met14* cir°) was used for all yeast transformations and grown in YPD (yeast extract, peptone, dextrose) medium supplemented with adenine. S. cerevisiae spheroplasts were prepared using previously described methods with the following modifications (16). Overnight cultures were grown to an optical density at 600 nm (OD_600_) of 1.8 to 2.5 and kept overnight at 4°C in 1 M sorbitol. After the cells were harvested, they were resuspended in 10 ml of SPE solution (1 M sorbitol, 0.01 M sodium phosphate, 0.01 M Na_2_EDTA) with 20 μl of β-mercaptoethanol and 20 μl of zymolyase solution. The cells were finally resuspended in 4 ml of STC solution (1 M sorbitol, 0.01 M Tris-HCl, 0.01 M CaCl_2_). DNA for transformation-associated recombination (TAR) cloning or assembly was added to 200 μl of spheroplasts, followed by the addition of 900 μl of 20% PEG solution (20% PEG 8000, 10 mM CaCl_2_, 10 mM Tris-HCl). The suspension was incubated at room temperature for 20 min, the PEG solution was removed, and the cells were then incubated at 30°C for 30 min in SOS medium (1 M sorbitol, 6.5 mM CaCl_2_, 0.25% yeast extract, 0.5% peptone). Transformed yeast spheroplasts were plated with selective top agar with sorbitol.

### TAR cloning, screening, and processing of HCMV DNA fragments.

Vectors were first PCR amplified using pCC1BAC-his3 as the template with Phusion (NEB) or Q5 (NEB) DNA polymerase. This plasmid contains bacterial artificial chromosome (BAC) and yeast centromeric plasmid (YCp) sequences for replication in E. coli and yeast. The primers add an I-SceI or PacI restriction site, flanked by 40 bp of HCMV homology, to each end of the vector backbone. PCR products were digested with DpnI (NEB) prior to transformation.

Viral DNA, which was isolated from Toledo P7 as described above, was sheared by pipetting. Six hundred nanograms of viral genomic DNA was cotransformed with 20 ng of the appropriate vector into yeast spheroplasts. Transformants were patched on synthetic dropout medium without histidine and supplemented with adenine (SD-HIS) plates and after sufficient growth were selected and transferred into 20 μl of 25 mM NaOH and incubated at 95°C for 30 min to lyse yeast cells. PCR on DNA from lysed yeast using the appropriate detection primers (see Table S1 in the supplemental material) was used to confirm the correct junction of the vector and HCMV fragment on each side.

Positive TAR clone candidates from yeast patches or 3 ml of liquid cultures were resuspended in 500 μl of water containing 5 μl of zymolyase 20T (MP Bio) (10 mg/ml) and 0.5 μl of β-mercaptoethanol (14.2 M) and incubated at 37°C for 1 h. Fifty microliters of 2% SDS was added, and the solution was incubated for 15 min at 70°C, followed by the addition of 50 μl of 5 M potassium acetate and incubation on ice for 5 min. After clarification, the DNA in the supernatant was precipitated with isopropanol and resuspended in 50 μl of Tris-EDTA (TE) buffer. Isolated DNA was then electroporated into E. coli EPI300 (Epicentre) or DH10B (Thermo Fisher) competent cells. DNA was purified from positive transformants for restriction enzyme analysis and sequencing.

### Assembly of complete HCMV genomes using TAR in yeast.

Cultures of all 16 TAR clones in E. coli were grown up, and DNA was isolated with PureLink HiPure Plasmid Midiprep kit (Thermo Fisher). Prior to assembly, the clones were digested with I-SceI and heat killed to release the HCMV fragments. Assembly of HCMV genomes was performed by TAR assembly in yeast in two steps. First, half genomes (TAR01 to TAR08 and TAR09 to TAR16) were assembled as follows. Vector DNA was amplified by PCR using pCCIBAC-ura3 as the template and either Con01R (R stands for reverse)and Con08F (F stands for forward) or CMV_1R and CMV_8F (Table S1) as primers for TAR01 to TAR08 or Con09R and Con16F or CMV_9R and CMV_16F (Table S1) for TAR09 to TAR16. For assembly of each half genome, 10 ng of vector DNA and 100 ng each of all other DNA fragments was added to spheroplasts. Transformants were patched on plates containing synthetic dropout medium without uracil and supplemented with adenine, and DNA was screened by PCR for the appropriate junctions with the respective detection primers (Table S1) using NaOH lysis. Positive half genomes were transformed into E. coli EPI300 competent cells and screened by PCR to confirm the appropriate junctions. For assembly of the full genome, the half genomes were processed with I-SceI or PacI endonuclease as described above to release the fragments. Vector DNA was amplified by PCR using pCCIBAC-his3 as the template and Con01R and Con16F or CMV_1R and CMV_16F as primers (Table S1). Ten nanograms of vector DNA and 100 ng each of the half genomes was added for assembly to spheroplasts. Transformants were patched onto SD-HIS plates, and DNA was screened by PCR for all of the appropriate junctions with the respective detection primers (Table S1) using NaOH lysis methods previously mentioned. As before, positive clones were transformed into E. coli EPI300 competent cells as described above and screened by PCR to confirm the appropriate junctions.

### Synthesis and purification of Cas9 and chimeric synthetic guide RNAs.

A single colony of E. coli Alpha-Select Gold Efficiency (Bioline) harboring the pJExpress404-FLAG-Cas9-His construct was cultured overnight in 50 ml LB supplemented with 100 μg/ml carbenicillin (Teknova). The starter culture was diluted into 1 liter of antibiotic-supplemented LB broth and shaken at 220 rpm at 37°C to an OD_600_ of 0.6. The culture was then cooled to 20°C, and isopropyl-β-d-thiogalactopyranoside (IPTG) was added to a final concentration of 0.5 mM to induce protein expression. Cas9 protein was induced overnight in a refrigerated incubator at 20°C under shaking (220 rpm). Cell pellets were washed once with phosphate-buffered saline (PBS), pH 7.4, and then resuspended in 60-ml volumes of xTractor buffer supplemented with EDTA-free protease inhibitor (Clontech), 2 mg lysozyme (Sigma-Aldrich), and 625 U Benzonase (Millipore). Cell suspensions were then gently rotated for 2 h at 4°C prior to mechanical lysis in a Microfluidizer M-110L (Microfluidics). Cell debris was pelleted at 8,000 × *g* for 1 h, and recombinant protein was purified from the supernatant using a HisTalon column (Clontech) according to the manufacturer’s instructions. Purified protein was stored at −80°C in Cas9 buffer (150 mM KCl, 20 mM HEPES [pH 7.4], 1 mM dithiothreitol [DTT], 10% glycerol) at a concentration of 4.5 mg/ml. The Cas9-targeting chimeric synthetic guide RNAs (sgRNAs) were designed, *in vitro* synthesized, and purified as described previously (42).

### Modification of Toledo-F TAR01 and TAR07.

Previously assembled HCMV Toledo-F fragments TAR01 and TAR07 contained within the E. coli-yeast shuttle pCC1BAC_HIS3 were purified using the QIAprep Spin Miniprep kit (Qiagen). Plasmid-borne genomic fragments were then independently digested with Cas9 in conjunction with two sgRNAs (fragment 1, 5′-CAAUGGACUGGCGAUUUAGUUUUAGAGCUAGAAAUAGCAAGUUAAAAUAAGGCUAGUCCGUUAUCAACUUGAAAAAGUGGCACCGAGUCGGUGCUUUUUUU-3′ and 5′-GAUGCGAUCGCAGUUACGGUUUUAGAGCUAGAAAUAGCAAGUUAAAAUAAGGCUAGUCCGUUAUCAACUUGAAAAAGUGGCACCGAGUCGGUGCUUUUUUU-3′ and fragment 7 5′-UGUGGAAUUCCGGACAUAGUUUUAGAGCUAGAAAUAGCAAGUUAAAAUAAGGCUAGUCCGUUAUCAACUUGAAAAAGUGGCACCGAGUCGGUGCUUUUUUU-3′ and 5′-UUACGUAUACCGGAUGCUGUUUUAGAGCUAGAAAUAGCAAGUUAAAAUAAGGCUAGUCCGUUAUCAACUUGAAAAAGUGGCACCGAGUCGGUGCUUUUUUU-3′ [Cas9 targeting sequences are underlined]), liberating DNA fragments of roughly 350 bp and 70 bp, respectively. One-hour digestions were performed at 37°C in 50-μl volume and contained 500 ng of plasmid DNA, 1 μg each gRNA, 10 mM spermidine (Sigma-Aldrich), 10 mM MgCl_2_ (Ambion), 20 mM HEPES, 150 mM KCl, 0.5 mM DTT, 0.1 mM EDTA, and 3.6 μg of Cas9 protein. Following digestion, Cas9 was heat inactivated at 80°C for 10 min, and digested DNA was extracted with UltraPure phenol-chloroform-isoamyl alcohol (25:24:1, vol/vol; pH 8), precipitated with ethanol, and resuspended in sterile water. DNA fragments used to generate Toledo-P insert fragments 1 and 7 were obtained from *de novo*-synthesized gblocks (IDT). The Toledo-P fragment 1 insert encoded a 279-bp insertion not found in the Toledo-F sequence. Conversely, the Toledo-P fragment 7 insert removed a 52-bp insert found only in Toledo-F. Both DNA inserts contained 20- to 100-bp overlap sequences homologous to regions upstream and downstream of the Cas9 digestion sites to facilitate *in vitro* assembly. Synthesized insert fragments were mixed with corresponding digested products at a 3:1 molar ratio (insert-vector) and covalently joined via standard Gibson Assembly reaction (SGI-DNA). Following assembly, constructs were transformed into E. coli TransforMax EPI300 electrocompetent cells (Epicentre) and plated on selective media. Transformants were verified to contain new inserts using PCR and Sanger sequencing prior to final confirmation of the full Toledo-P genome sequence by next-generation sequencing (NGS).

### Modification of Toledo-F TAR013.

NGS sequencing data indicated that the Toledo-P genome contained a large 14.3-kb genomic inversion flanked by two small insertions (14 and 23 bp). This large inversion was entirely contained within the previously generated Toledo-F genomic fragment 13. Utilizing primers 5′-GCAAAGTGAACGACAAGGCGCAGTACCTGCTG-3′ and 5′-CCTAGTAACACTCGTCCGACACTTCCACCATCTCCAGC-3′ (IDT), the ends of genomic fragment 13 backbone were PCR amplified together with the E. coli-yeast shuttle pCC1BAC_HIS3 vector. In a second PCR, the 14.3-kb inverted sequence was amplified with Ultramer oligonucleotides 5′-CTCTCCAGGTACTGATCCAGGCCCACGATCCGGGTTATCTTGTCGTATTCCAGGTTGATCCATCGATAGGGAACGCTGCCAGCGGCGCCCAGCAGGTACTGCGCCTTGTCGTTCACTTTGCCGCAGCGTATTCGCCCGTCAGCTTCGAGGTATAACCTACAACACGGAGGGGAAGGGGGGTACAAAACGTGAAATTAGAC-3′ and 5′-GAGACGACGCCGCTGGTAGAGGATGCCGAACCGCCGGCCGAGCTGGAGATGGTGGAAGTGTCGGACGAGTGTTACTAGGAGATCGCCGCGGCCGATGGGCGCCGGCGGACGTGACTCGGCAGCCGCTGTAGGGATAAATAGTGCGATGGCGTTTGTGG-3′, generating an inverted sequence with the indicated terminal inserts flanked by 121-bp overlap sequences homologous to regions at the ends of the first amplicon. Both PCRs were performed with the KOD Xtreme Hot Start DNA polymerase (Millipore) using the Toledo-F genomic fragment 13 as the template and according to the manufacturer’s specifications. The amplicons were digested with DpnI (NEB) and purified using the QIAquick PCR purification kit (Qiagen). Following purification, amplicons were mixed at an equal molar ratio and covalently joined via standard Gibson Assembly reaction (SGI-DNA). Following assembly, constructs were transformed into E. coli TransforMax EPI300 electrocompetent cells (Epicentre) and plated on selective media. Transformants were verified to contain new inserts using PCR and Sanger sequencing prior to final confirmation of the full Toledo-P genome sequence by NGS.

### Engraftment and infection of humanized mice.

All animal studies were carried out in strict accordance with the recommendations of the American Association for Accreditation of Laboratory Animal Care (AAALAC). The protocol was approved by the Institutional Animal Care and Use Committee (number IS00003498) at the Oregon Health & Science University. NOD-*scid* IL2Rγ_c_^null^ (NSG) mice were maintained at a pathogen-free facility at Oregon Health & Science University in accordance with procedures approved by the Institutional Animal Care and Use Committee. Both sexes of animals were used. Humanized mice were generated as previously described (30). The animals (12 to 14 weeks after engraftment) were treated with 25 ng/mouse lipopolysaccharide (LPS), and after 6 h, they were infected via intraperitoneal injection of human dermal fibroblasts previously infected with HCMV TR, Toledo-P, or Toledo-F at approximately 5 × 10^5^ PFU per mouse. A control group of engrafted mice were mock infected using uninfected fibroblasts. At 4 weeks postinfection, the infected mice were split into two groups, and one group was treated with 100 μl of granulocyte colony-stimulating factor (G-CSF) (300 mg/ml; Amgen) using a subcutaneous micro-osmotic pump (1007D; Alzet) and 125 μg AMD3100 [1,1′-[1,4-phenylenebis(methylene)]bis-1,4,8,11-tetra-azacyclotetradecane octahydrochloride or plerixafor] administered intraperitoneally to mobilize hematopoietic progenitor cells (HPCs). The remaining mice serve as a direct comparison for the effects of virus reactivation and dissemination that follow HPC mobilization. One week after HPC mobilization, the mice were sacrificed, their organs were harvested, and samples for PCR were frozen in RNAlater solution and stored at −80°C for subsequent analysis.

### Quantitative PCR for viral genomes.

Total DNA was extracted from approximately 1-mm^2^ sections of mouse spleen or liver using the DNAzol kit (Life Technologies). HCMV genomes were analyzed using quantitative PCR (TaqMan) performed on 1 µg of total DNA and using TaqMan FastAdvance PCR master mix (Applied Biosystems) according to the manufacturer’s instructions. Primers and a probe recognizing HCMV UL141 were used to quantify HCMV genomes (probe, CGAGGGAGAGCAAGTT; forward primer, 5′-GATGTGGGCCGAGAATTATGA; reverse primer, 5′-ATGGGCCAGGAGTGTGTCA). The probe contains a 5′ FAM (6-carboxyfluorescein) reporter molecule and a 3′ quencher molecule (Applied Biosystems). The reaction was initiated using TaqMan Fast Advanced master mix (Applied Biosystems) activated at 95°C for 10 min followed by 40 cycles (1 cycle consists of 15 s at 95°C and 1 min at 60°C) using a StepOnePlus TaqMan thermocycler. Results were analyzed using ABI StepOne software. Data were analyzed using the statistical program GraphPad Prism 5. Statistical significance was determined using two-way analysis of variance, followed by Bonferroni’s posttest correction. A *P* value of <0.05 or lower was considered significant.

### Accession number(s).

The genome sequences of P7 Toledo-F, P7 Toledo-P as well as assembled Toledo-F and Toledo-P have been submitted to GenBank under accession numbers MF783090 to MF783093.
