# Supplementary material for: Cloning, Assembly, and Modification of the Primary Human Cytomegalovirus Isolate Toledo by Yeast-Based Transformation-Associated Recombination
Source: mSphere. 2017 Oct 4;2(5):e00331-17. doi: 10.1128/mSphereDirect.00331-17 (PMC5628293; doi:10.1128/mSphereDirect.00331-17)
Supplement: FIG S1 [file sph005172375sf1.pdf]

A

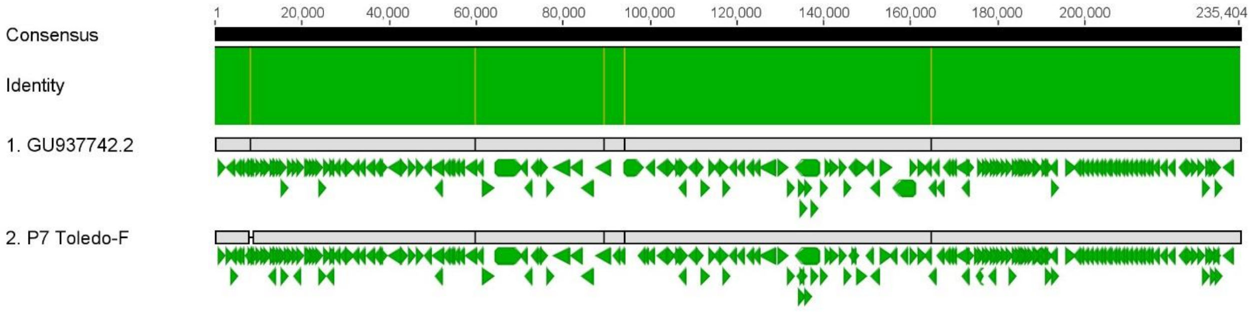

B

Sequence aligned to	Coordinate	Type
GU937742.2	8274	insertion of G in non-coding region (likely homopolymer)
GU937742.2	59592	SNP A to C causes L to W in UL45
GU937742.2	89517	SNP G to C synonymous mutation G to G
GU937742.2	94188	SNP T to C synonymous mutation P to P
GU937742.2	94227	SNP T to G synonymous mutation A to A
GU937742.2	164390	SNP A to G synonymous mutation S to S

Figure S1
