# Supplementary figures and images for: Cloning, Assembly, and Modification of the Primary Human Cytomegalovirus Isolate Toledo by Yeast-Based Transformation-Associated Recombination
Source: mSphere. 2017 Oct 4;2(5):e00331-17. doi: 10.1128/mSphereDirect.00331-17 (PMC5628293; doi:10.1128/mSphereDirect.00331-17)

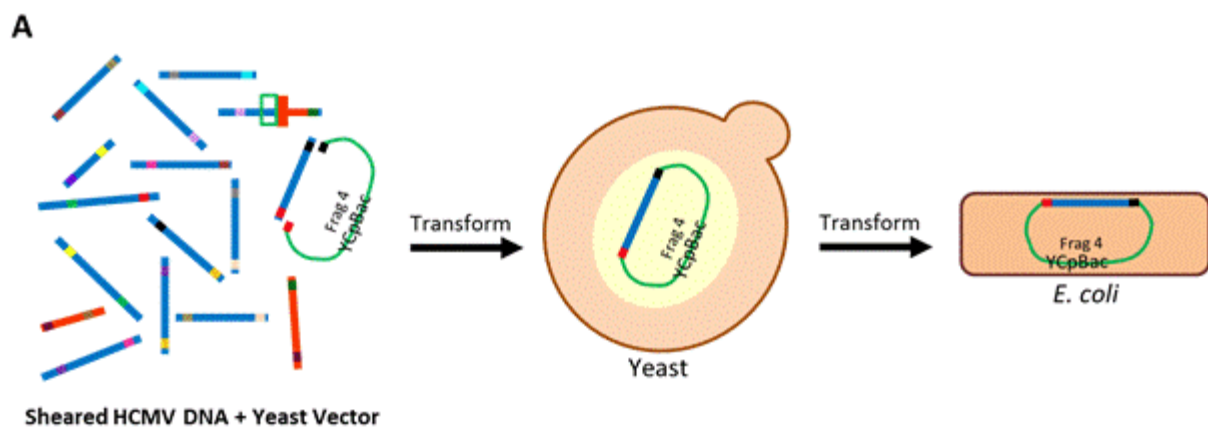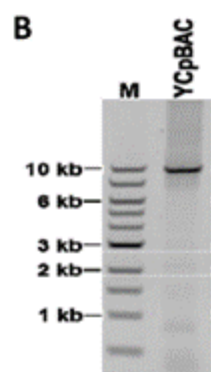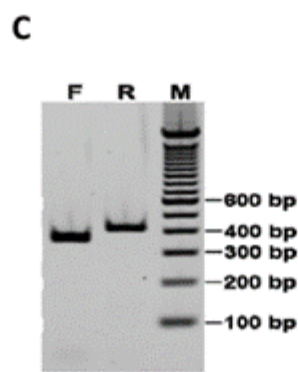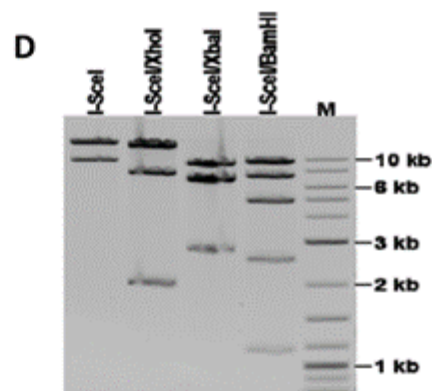

**Figure S2**

Supplement: FIG S2 [file sph005172375sf2.pdf]
