# Supplementary material for: Cloning, Assembly, and Modification of the Primary Human Cytomegalovirus Isolate Toledo by Yeast-Based Transformation-Associated Recombination
Source: mSphere. 2017 Oct 4;2(5):e00331-17. doi: 10.1128/mSphereDirect.00331-17 (PMC5628293; doi:10.1128/mSphereDirect.00331-17)
Supplement: TABLE S1 [file sph005172375st3.docx]

**Table S1. Primers used in this study.**

| **Name** | **Sequence** | **Description** |
| --- | --- | --- |
| Con01F | AGGTGATCCAGTTACCCTCGGTAGTGGACATGGTTATCATCCAGGtagggataacagggtaatgatcctctagagtcgacctg | Forward to amplify TAR vector 01 from pCC1BAC/YCp (blue) and add I-SceI recognition site (red) as well as HCMV homology (capitals) |
| Con01R | AACACCCCCCCGCCCCTCGGGGACCCAGCACACGGCCCGGAATGGtagggataacagggtaatcgggtaccgagctcgaattc | Reverse to amplify TAR vector 01 from pCC1BAC/YCp (blue) and add I-SceI recognition site (red) as well as HCMV homology (capitals) |
| CMV_1R | CCCCCCGCCCCTCGGGGACCCAGCACACGGCCCGGAATGGttaattaagggccgtcgaccaattctca | Reverse to amplify TAR vector 01 from pCC1BAC/YCp (blue) and add PacI recognition site (red) as well as HCMV homology (capitals) |
| Con02F | CGTCGTCGTCACTCGTGGCGTCACAGTCAACGGTGCTGACGTCCTtagggataacagggtaatgatcctctagagtcgacctg | Forward to amplify TAR vector 02 from pCC1BAC/YCp (blue) and add I-SceI recognition site (red) as well as HCMV homology (capitals) |
| Con02R | TGGATCACCTACTCTGACAGTGACCTCCATACAGGTTTCGGAATAtagggataacagggtaatcgggtaccgagctcgaattc | Reverse to amplify TAR vector 02 from pCC1BAC/YCp (blue) and add I-SceI recognition site (red) as well as HCMV homology (capitals) |
| Con03F | CATAACCTCAGCGGGGTATGAGCTTTCCTGTTACTTTATTCAGAAtagggataacagggtaatcgatcctctagagtcgacctg | Forward to amplify TAR vector 03 from pCC1BAC/YCp (blue) and add I-SceI recognition site (red) as well as HCMV homology (capitals) |
| Con03R | GACGACGACGCTGCTTGGGGCATAAAACACCGACGCTACTTCCCGtagggataacagggtaatcgggtaccgagctcgaattc | Reverse to amplify TAR vector 03 from pCC1BAC/YCp (blue) and add I-SceI recognition site (red) as well as HCMV homology (capitals) |
| Con04F | ACGGCGACTCGGGACGCCAACTGACGACGCCGCCACCACTCGTAAtagggataacagggtaatgatcctctagagtcgacctg | Forward to amplify TAR vector 04 from pCC1BAC/YCp (blue) and add I-SceI recognition site (red) as well as HCMV homology (capitals) |
| Con04R | TGAGGTTATGATGCGATTGTGAGAGTCGATGGGGGATTTTTGTGGtagggataacagggtaatcgggtaccgagctcgaattc | Reverse to amplify TAR vector 04 from pCC1BAC/YCp (blue) and add I-SceI recognition site (red) as well as HCMV homology (capitals) |
| Con05F | GACGATGAGCGACTCGTTCACCTTAAGCACATTGAACTCACCTACtagggataacagggtaatgatcctctagagtcgacctg | Forward to amplify TAR vector 05 from pCC1BAC/YCp (blue) and add I-SceI recognition site (red) as well as HCMV homology (capitals) |
| Con05R | GAGTCGCCGTCTCCCGACGGCACGCCGTCTGTGCTGTCGTTGACGtagggataacagggtaatcgggtaccgagctcgaattc | Reverse to amplify TAR vector 05 from pCC1BAC/YCp (blue) and add I-SceI recognition site (red) as well as HCMV homology (capitals) |
| Con06F | TGAGCGCCGAAAAGACGGCCACAAAGTCGCTTTTGCCGTGCGCGCtagggataacagggtaatgatcctctagagtcgacctg | Forward to amplify TAR vector 06 from pCC1BAC/YCp (blue) and add I-SceI recognition site (red) as well as HCMV homology (capitals) |
| Con06R | AGATCAAGCCCTCGGCCTACGGAGTGCTGACGAAGTGCGTGGTGCtagggataacagggtaatcgggtaccgagctcgaattc | Reverse to amplify TAR vector 06 from pCC1BAC/YCp (blue) and add I-SceI recognition site (red) as well as HCMV homology (capitals) |
| Con07F | AGTGCACGCAGACCGTGTCGGGCAGACGCGCACGTTCGCGGAACGtagggataacagggtaatgatcctctagagtcgacctg | Forward to amplify TAR vector 07 from pCC1BAC/YCp (blue) and add I-SceI recognition site (red) as well as HCMV homology (capitals) |
| Con07R | TCGGCGCTCAACAAGTTTGTGGACGACGAGGCCCTGGGTTTCGTTtagggataacagggtaatcgggtaccgagctcgaattc | Reverse to amplify TAR vector 07 from pCC1BAC/YCp (blue) and add I-SceI recognition site (red) as well as HCMV homology (capitals) |
| Con08F | TTGTGTCGCGCCACCACCTCGGCGCGATGCGTGTAAACCGAAAAGtagggataacagggtaatgatcctctagagtcgacctg | Forward to amplify TAR vector 08 from pCC1BAC/YCp (blue) and add I-SceI recognition site (red) as well as HCMV homology (capitals) |
| CMV_8F | TCGCGCCACCACCTCGGCGCGATGCGTGTAAACCGAAAAGttaattaagtccacataaccgtgcgcaa | Forward to amplify TAR vector 08 from pCC1BAC/YCp (blue) and add PacI recognition site (red) as well as HCMV homology (capitals) |
| Con08R | TGCGTGCACTACGTCTACCTGGCCTACCGCACGGCGCTGGCACGCtagggataacagggtaatcgggtaccgagctcgaattc | Reverse to amplify TAR vector 08 from pCC1BAC/YCp (blue) and add I-SceI recognition site (red) as well as HCMV homology (capitals) |
| Con09F | TCTGCTCCAGAAGATACTCGATGGGGTCGTGGCTCAGCTTGATGGtagggataacagggtaatgatcctctagagtcgacctg | Forward to amplify TAR vector 09 from pCC1BAC/YCp (blue) and add I-SceI recognition site (red) as well as HCMV homology (capitals) |
| Con09R | CGCGACACAATCCGTACCCGCACCTACGACGCTTGCCGGACAACGtagggataacagggtaatcgggtaccgagctcgaattc | Reverse to amplify TAR vector 09 from pCC1BAC/YCp (blue) and add I-SceI recognition site (red) as well as HCMV homology (capitals) |
| CMV_9R | CACAATCCGTACCCGCACCTACGACGCTTGCCGGACAACGttaattaagggccgtcgaccaattctca | Reverse to amplify TAR vector 09 from pCC1BAC/YCp (blue) and add PacI recognition site (red) as well as HCMV homology (capitals) |
| Con10F | CGGCTTTTCGCGAGCGACCGGCCGGCGGCGATCGTCGCTGGCTGTtagggataacagggtaatgatcctctagagtcgacctg | Forward to amplify TAR vector 10 from pCC1BAC/YCp (blue) and add I-SceI recognition site (red) as well as HCMV homology (capitals) |
| Con10R | CTGGAGCAGATTCAGAACCTGCACCGCGTCACGCTGGCCGAAGGCtagggataacagggtaatcgggtaccgagctcgaattc | Reverse to amplify TAR vector 10 from pCC1BAC/YCp (blue) and add I-SceI recognition site (red) as well as HCMV homology (capitals) |
| Con11F | TCCGGATCACATGGTTACTCAGCGTCTGCCAGCCTAAGTGACGGTtagggataacagggtaatgatcctctagagtcgacctg | Forward to amplify TAR vector 11 from pCC1BAC/YCp (blue) and add I-SceI recognition site (red) as well as HCMV homology (capitals) |
| Con11R | CGAAAAGCCGTGGCATTGAGACGCACGGCGCCGCCGCCGGCCTCGtagggataacagggtaatcgggtaccgagctcgaattc | Reverse to amplify TAR vector 11 from pCC1BAC/YCp (blue) and add I-SceI recognition site (red) as well as HCMV homology (capitals) |
| Con12F | GTCGGCGACAGAAATCTCAAAACGCGTATTTCGGACAAACACACAtagggataacagggtaatgatcctctagagtcgacctg | Forward to amplify TAR vector 12 from pCC1BAC/YCp (blue) and add I-SceI recognition site (red) as well as HCMV homology (capitals) |
| Con12R | GTGATCCGGAGGCTGTCAGAGCGCCGGGAGCATCTGGTGTTCATGtagggataacagggtaatcgggtaccgagctcgaattc | Reverse to amplify TAR vector 12 from pCC1BAC/YCp (blue) and add I-SceI recognition site (red) as well as HCMV homology (capitals) |
| Con13F | TTTAAAAACCATCATCGACGGCCGTTATAAAGCCACCCGGACACGtagggataacagggtaatgatcctctagagtcgacctg | Forward to amplify TAR vector 13 from pCC1BAC/YCp (blue) and add I-SceI recognition site (red) as well as HCMV homology (capitals) |
| Con13R | TGTCGCCGACTAAATTCATGTCGCGCGATAGTGGTGTTTATCGCCtagggataacagggtaatcgggtaccgagctcgaattc | Reverse to amplify TAR vector 13 from pCC1BAC/YCp (blue) and add I-SceI recognition site (red) as well as HCMV homology (capitals) |
| Con14F | TGACAGGACATAGGCCTGGATTACCACGGTGCGATCGAAACACAGtagggataacagggtaatgatcctctagagtcgacctg | Forward to amplify TAR vector 14 from pCC1BAC/YCp (blue) and add I-SceI recognition site (red) as well as HCMV homology (capitals) |
| Con14R | TGATGGTTTTTAAACCGGGACGACGGTGGTTCACGTGAAGTACCAtagggataacagggtaatcgggtaccgagctcgaattc | Reverse to amplify TAR vector 14 from pCC1BAC/YCp (blue) and add I-SceI recognition site (red) as well as HCMV homology (capitals) |
| Con15F | CGGCAACAGATGAGCTTTGACGCCGCCTGTTTGGCGGCGGTAATGtagggataacagggtaatgatcctctagagtcgacctg | Forward to amplify TAR vector 15 from pCC1BAC/YCp (blue) and add I-SceI recognition site (red) as well as HCMV homology (capitals) |
| Con15R | TGTCCTGTCATCCATGCTCTGCGTCTGGTGTACGGGCCTAGCCTGtagggataacagggtaatcgggtaccgagctcgaattc | Reverse to amplify TAR vector 15 from pCC1BAC/YCp (blue) and add I-SceI recognition site (red) as well as HCMV homology (capitals) |
| Con16F | CGCGCCCCCGACACACCCCGAACGGCGCCGGTGCGGGACAGGGCTtagggataacagggtaatgatcctctagagtcgacctg | Forward to amplify TAR vector 16 from pCC1BAC/YCp (blue) and add I-SceI recognition site (red) as well as HCMV homology (capitals) |
| CMV_16F | CCCCGACACACCCCGAACGGCGCCGGTGCGGGACAGGGCTttaattaagtccacataaccgtgcgcaa | Forward to amplify TAR vector 16 from pCC1BAC/YCp (blue) and add PacI recognition site (red) as well as HCMV homology (capitals) |
| Con16R | TCTGTTGCCGCGAGCCGCTGACGCCGTTGGGATACGCTGTTATTTtagggataacagggtaatcgggtaccgagctcgaattc | Reverse to amplify TAR vector 16 from pCC1BAC/YCp (blue) and add I-SceI recognition site (red) as well as HCMV homology (capitals) |
| Det01F | AAGAATCAACGCCAAGGAAA | Forward to detect Toledo-F TAR01 |
| Det01R | CCTCTTTTCCGCGTCGTTTC | Reverse to detect Toledo-F TAR01 |
| Det02F | TCTTTGGGCCAGACCAG | Forward to detect Toledo-F TAR02 |
| Det02R | CTGGTCGTTGTCGTTCTGGA | Reverse to detect Toledo-F TAR02 |
| Det03F | TTGCACTGCCTCATTAATCC | Forward to detect Toledo-F TAR03 |
| Det03R | ACTTGCTGCGCAACATCAAA | Reverse to detect Toledo-F TAR03 |
| Det04F | CCGTGACAATGACACTTTTT | Forward to detect Toledo-F TAR04 |
| Det04R | TCACCGCTCGGAGAAAACAA | Reverse to detect Toledo-F TAR04 |
| Det05F | CTGTGACTGCTTACGGTG | Forward to detect Toledo-F TAR05 |
| Det05R | CTTATGAATCCGGCTGACGC | Reverse to detect Toledo-F TAR05 |
| Det06F | AATGGATTGAAAATGCTGCG | Forward to detect Toledo-F TAR06 |
| Det06R | GGCGTATCCTCAAGTTGGGT | Reverse to detect Toledo-F TAR06 |
| Det07F | TGGAGGTGCCGAAGAAG | Forward to detect Toledo-F TAR07 |
| Det07R | GTTACAACGTCGACGCCAAG | Reverse to detect Toledo-F TAR07 |
| Det08F | TGTGAGAGTCCAAAACCCA | Forward to detect Toledo-F TAR08 |
| Det08R | GCTGACCATGGCCTACTACC | Reverse to detect Toledo-F TAR08 |
| Det09F | CCTTGGGGAGTTTTACGC | Forward to detect Toledo-F TAR09 |
| Det09R | CTGACCGTAACGCCGAACT | Reverse to detect Toledo-F TAR09 |
| Det10F | GTGCTTTTTCGTGGTTTCG | Forward to detect Toledo-F TAR10 |
| Det10R | GTGGAGGGCAACACTAACCA | Reverse to detect Toledo-F TAR10 |
| Det11F | CATCAATCACCCACAGAGTC | Forward to detect Toledo-F TAR11 |
| Det11R | ACCGCATAAGTGTGGAGTCG | Reverse to detect Toledo-F TAR11 |
| Det12F | GAAACTGCGATATTTGCGAC | Forward to detect Toledo-F TAR12 |
| Det12R | CTGGAGCTATCTGTGTCCGC | Reverse to detect Toledo-F TAR12 |
| Det13F | GACCTTCGTGCGTTGTC | Forward to detect Toledo-F TAR13 |
| Det13R | GCTACTGCGTTCTCCCACAA | Reverse to detect Toledo-F TAR13 |
| Det14F | TGTAGCAATTTGACGGTGAG | Forward to detect Toledo-F TAR14 |
| Det14R | CCATCTCCTGATGGCGTTGT | Reverse to detect Toledo-F TAR14 |
| Det15F | GGTGTGGTGTCATTGAGAAA | Forward to detect Toledo-F TAR15 |
| Det15R | AAGATGAAGACACCCTGCGG | Reverse to detect Toledo-F TAR15 |
| Det16F | CACACTTGTTGTTCCCTCC | Forward to detect Toledo-F TAR16 |
| Det16R | ATTTGGCTCGGCTTCTGTCA | Reverse to detect Toledo-F TAR16 |
| RCO493 | CCAAGCTATTTAGGTGAGAC | Detect pCC1BAC backbone sequence, for use with F Det primers |
| RCO495 | ACGACGGCCAGTGAATTG | Detect pCC1BAC backbone sequence, for use with R Det primers |
| MODTAR13 Primer 1 | CCTAGTAACACTCGTCCGACACTTCCACCATCTCCAGC | Forward to amplify MODTAR13 YCpBAC vector |
| MODTAR13 Primer 2 | GCAAAGTGAACGACAAGGCGCAGTACCTGCTG | Reverse to amplify MODTAR13 YCpBAC vector |
| MODTAR13 Primer 3 | GAGACGACGCCGCTGGTAGAGGATGCCGAACCGCCGGCCGAGCTGGAGATGGTGGAAGTGTCGGACGAGTGTTACTAGGAGATCGCCGCGGCCGATGGGCGCCGGCGGACGTGACTCGGCAGCCGCTGTAGGGATAAATAGTGC GATGGCGTTTGTGG | Forward to amplify and correct inverted UL128-133 region from Toledo-F TAR13 |
| MODTAR13 Primer 4 | CTCTCCAGGTACTGATCCAGGCCCACGATCCGGGTTATCTTGTCGTATTCCAGGTTGATCCATCGATAGGGAACGCTGCCAGCGGCGCCCAGCAGGTACTGCGCCTTGTCGTTCACTTTGCCGCAGCGTATTCGCCCGTCAGCTTCGAGGTATAACCTACAACACGGAGGGGAAGGGGGGTACAAAACGTGAAATTAGAC | Reverse to amplify and correct inverted UL128-133 region from Toledo-F TAR13 |
