# Supplementary material for: Cloning, Assembly, and Modification of the Primary Human Cytomegalovirus Isolate Toledo by Yeast-Based Transformation-Associated Recombination
Source: mSphere. 2017 Oct 4;2(5):e00331-17. doi: 10.1128/mSphereDirect.00331-17 (PMC5628293; doi:10.1128/mSphereDirect.00331-17)
Supplement: TABLE S2 [file sph005172375st4.docx]

**Table S2. Design of HCMV fragments.**

| TAR Fragment | Start Coordinate^*^ | End Coordinate^*^ | Length |
| --- | --- | --- | --- |
| 1 | 1 | 17596 | 17596 |
| 2 | 17517 | 28969 | 11453 |
| 3 | 28890 | 44474 | 15585 |
| 4 | 44395 | 60090 | 15696 |
| 5 | 60011 | 73935 | 13925 |
| 6 | 73827 | 89377 | 15551 |
| 7 | 89298 | 103591 | 14294 |
| 8 | 103512 | 119349 | 15838 |
| 9 | 119270 | 133605 | 14336 |
| 10 | 133526 | 148641 | 15116 |
| 11 | 148562 | 164196 | 15635 |
| 12 | 164117 | 175783 | 11667 |
| 13 | 175704 | 191529 | 15826 |
| 14 | 191454 | 206761 | 15308 |
| 15 | 206682 | 220627 | 13946 |
| 16 | 220548 | 235398 | 14851 |

^*^Coordinates based on the Toledo strain reference sequence (GenBank GU937742.1)
